# Light pollution is associated with earlier tree budburst across the United Kingdom

**DOI:** 10.1098/rspb.2016.0813

**Published:** 2016-06-29

**Authors:** Richard H. ffrench-Constant, Robin Somers-Yeates, Jonathan Bennie, Theodoros Economou, David Hodgson, Adrian Spalding, Peter K. McGregor

**Affiliations:** 1 Centre for Ecology and Conservation, University of Exeter, Penryn TR10 9EZ, UK; 2 Environment and Sustainability Institute, University of Exeter, Penryn TR10 9EZ, UK; 3 College of Engineering, Mathematics and Physical Sciences, University of Exeter, Exeter EX4 UY, UK; 4 Spalding Associates (Environmental) Ltd, 10 Walsingham Place, Truro TR1 2RP, UK; 5 Centre for Applied Zoology, Cornwall College Newquay, Newquay TR7 2LZ, UK

**Keywords:** light pollution, phenology, species interactions, tree budburst, temperature, urban heat islands

## Abstract

The ecological impact of night-time lighting is of concern because of its well-demonstrated effects on animal behaviour. However, the potential of light pollution to change plant phenology and its corresponding knock-on effects on associated herbivores are less clear. Here, we test if artificial lighting can advance the timing of budburst in trees. We took a UK-wide 13 year dataset of spatially referenced budburst data from four deciduous tree species and matched it with both satellite imagery of night-time lighting and average spring temperature. We find that budburst occurs up to 7.5 days earlier in brighter areas, with the relationship being more pronounced for later-budding species. Excluding large urban areas from the analysis showed an even more pronounced advance of budburst, confirming that the urban ‘heat-island’ effect is not the sole cause of earlier urban budburst. Similarly, the advance in budburst across all sites is too large to be explained by increases in temperature alone. This dramatic advance of budburst illustrates the need for further experimental investigation into the impact of artificial night-time lighting on plant phenology and subsequent species interactions. As light pollution is a growing global phenomenon, the findings of this study are likely to be applicable to a wide range of species interactions across the world.

## 1. Introduction

Most organisms have evolved for millions of years under predictable cycles of light and dark resulting from the Earth's rotation and orbit. Ambient light plays an important role in natural systems, acting as an abiotic cue organizing both daily and seasonal patterns in activity [1]. At higher latitudes, changes in day length are therefore an accurate indicator of the progression of the season, and specifically the onset of more favourable spring conditions [2]. However, the extent to which these fundamental light-driven processes are being influenced by light pollution is unclear. We wanted to assess whether UK-wide data on night-time lighting could be correlated with advances in tree budburst.

Vascular plants use phytochrome photoreceptors, sensitive to the red : far red ratio of light, to effectively determine the day length, and this ability assists them in timing key phenological events such as budburst, flowering and bud set, so that they coincide with favourable environmental conditions [1–3]. An experiment reducing the red : far red ratio of light at twilight advanced budburst in silver birch (*Betula pendula*) by approximately 4 days [4]. For many organisms, the accurate timing of such events has important fitness effects. Further, in multi-trophic systems, the period of optimal conditions is often governed in part by species phenology at the underlying trophic level [5]. This is well exemplified by the interaction between the Pendunculate oak tree (*Quercus robur*) host plant and its winter moth caterpillar (*Operophtera brumata*) herbivore, which has been extensively examined in the context of the likely impacts of anthropogenic climate change on phenology [5–7]. Oak trees are thought to use both temperature and photoperiod as abiotic cues, to unfurl their buds at a time that will maximize the length of the growing season, while at the same time reducing the risk of frost damage [2,8–10]. In turn, the winter moth herbivore is under pressure to match its egg hatch with the timing of budburst. Thus, if the eggs hatch too early, the larvae may face starvation, and if they hatch too late, they will be forced to eat less digestible, and better protected tannin-rich leaves [5,7,11]. A combination of photoperiod and temperature forcing is considered to be important for determining budburst phenology in most temperate trees, with the temperature forcing requirement for bud burst decreasing to a minimal value when accumulated winter chilling and/or increases in photoperiod have been detected [12]. Opportunistic, early successional species, and tree species that come into leaf earlier in the spring, tend to be more sensitive to temperature alone with little influence of photoperiod. Late-successional species, which also tend to break bud later in the spring, tend to have a more marked response to photoperiod [2,8]. Observations suggest that the leaf phenology of several urban tree species is altered in the direct vicinity of street lighting, both in terms of earlier budburst and later leaf fall [13].

Over the last 150 years, the natural night-time environment has been drastically altered by the proliferation of man-made artificial lighting. In 2001, it was estimated that almost a fifth of the Earth's land surface was polluted by light [14], and subsequently the amount of artificial light has been increasing at approximately 6% annually [15]. The increasingly large amount of artificial night-time lighting and the known importance of light to natural systems have led to widespread concern over the potential ecological impacts of light pollution [15–19]. Specifically, concern has been expressed about the potential of light pollution to disrupt trophic interactions through artificially altering the day length as perceived by living organisms [1,16,20]. Spring phenology including tree budburst is advanced in urban areas, and it is generally considered that the main cause is the urban ‘heat island’ (UHI) effect of enhanced temperature regimes [21–27]. However, experiments that artificially altered photoperiod have shown that budburst of a number of species of late-successional trees was delayed when the photoperiod was shortened [2,8]. The night-time light environment of urban and suburban areas is extremely heterogeneous, with light intensities varying across several orders of magnitude over horizontal and vertical distances of a few metres [13]. Here we therefore examined the hypothesis that increasing photoperiod, via artificial lighting, will hasten the earliest recorded date of budburst, and that this effect will be greater in late-budding than early budding species. To test this hypothesis, we analysed spatio-temporal data on budburst and satellite imagery of night-time lighting to investigate whether light pollution is correlated with budburst date. Strikingly, we find that earlier budburst is associated with night-time lighting, that the effect is larger in late- than early budding species, and that magnitude of this advance is likely to be too great to be explained by residual urban temperature effects alone.

## 2. Material and methods

### (a) Budburst data

Spatially referenced budburst data were collected from 1999 to 2011 by ‘citizen scientists’ and submitted to the UK phenology network (www.naturescalendar.org.uk). We used data from four available deciduous species: European sycamore (*Acer pseudoplatanus*, known as sycamore maple in North America), European beech (*Fagus sylvatica*), Pedunculate oak (*Q. robur*) and European ash (*Fraxinus excelsior*). Recorders were asked to note ‘budburst’ as the date when the colour of the new green leaves is just visible between the scales of the swollen or elongated bud; they were advised, if they were having difficulty in deciding when to record, to wait until the event was occurring in three plants of the same species within close proximity to each other, to record the trendsetters rather than the extraordinary. Each record from a single observer is georeferenced and was treated as a separate point observation, therefore even if several observers record at the same or nearby points (as is likely to be the case in more densely populated areas), then there should be no systematic bias towards earlier records, assuming that the distribution of recording effort and accuracy made by individual observers is independent of recorder density. The potential recorder error within the data collection protocol was deemed unlikely to be problematic in the present analyses, as there is no reason to expect that the distribution of recording effort and accuracy of individual observers is affected by observer density or the amount of artificial night-time lighting.

### (b) Light pollution data and calibration

The global dataset of annual night-time satellite images for 1999–2011 from the Defense Meteorological Satellite Program's Operational Linescan System (DMSP OLS) was used to quantify the amount of artificial light at the locations of the spatially referenced budburst dates. These data are produced and made publicly available by the NOAA National Geophysical Data Centre [28] and have previously been used to map the extent of light pollution [14,29,30]. These satellite images depict a global, cloud-free composite of stable night-time light at approximately 1 km resolution, re-sampled from data at a resolution of approximately 2.7 km. Each pixel is represented by a value of 0–63; a value of zero represents areas of relative darkness, whereas brightly lit urban areas usually saturate at a value of 63. (Given the coarse resolution of these data, a spatially referenced budburst date within a bright pixel, for example, will not necessarily be located in a bright area; it is just assumed to be more likely to be.) These data will here be referred to as either DMSP data or DMSP value.

Accurate inter-annual comparisons of the DMSP data are difficult because the data have been collected by multiple satellites with a lack of onboard intercalibration between the satellite sensors and the gain control of their optical sensors is changed continually to generate consistent imagery of clouds. This means that a specific pixel value in a given year may not represent the same actual level of brightness as a pixel of the same nominal value in another year. In addition, there are inaccuracies with the geolocation of the DMSP data which result in apparent differences in the location of pixels between years; up to 3 pixels (approx. 3 km) between some years [31]. In order to compare images between years, the geolocation errors must therefore be rectified and the images intercalibrated. In this study, correction of geolocation errors and intercalibration of images followed the methods described in a previous study [31]. Intercalibrated DMSP data were resampled, using bilinear interpolation, to a 5 km grid to match the resolution of the temperature data.

### (c) Gridded temperature data

Owing to the increased amount of artificial light in urban areas, and the fact that urban areas are known to be warmer than surrounding rural areas because of the UHI effect [32], we anticipated that temperature would positively covary with the amount of artificial light. To control for this potential covariance, 5 × 5 km gridded mean monthly air temperature data were incorporated into the analysis. This gridded air temperature data cover the majority of the UK and were created using weather station data, through an interpolation process that takes into account topographical, coastal and urban features [33] (www.metoffice.gov.uk/climatechange/science/monitoring/ukcp09/). For the present analysis, a new 5 × 5 km gridded dataset of average spring air temperatures was created for 1999–2011 from the monthly gridded temperatures. This was performed by averaging the temperatures for February to April, as the timing of first leaf date is known to strongly correlate with temperatures within this period [34].

### (d) Spatial matching and statistical analysis

**Figure 1.**
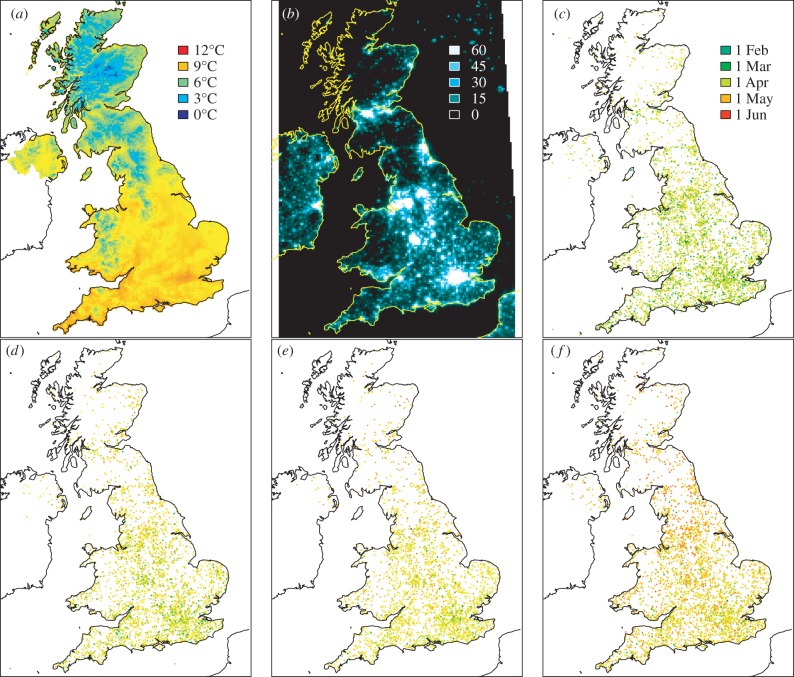
(*a*) Average spring temperatures in 2011, (*b*) DMSP night-time lights in 2011, (*c–f*) locations of budburst data for all years, for (in order of budburst) sycamore (*c*), beech (*d*), oak (*e*) and ash (*f*).

Budburst data were spatially matched with both resampled DMSP light pollution values and mean air temperature values within years, giving 11 968 data points for *Acer*, 10 061 points for *Fagus*, 8908 data points for *Quercus* and 10 899 for *Fraxinus*. The light and temperature data for each budburst sampling point were extracted using bilinear interpolation from the 5 km resolution grids. The budburst data were transformed from British National Grid to WGS1984 before extracting the DMSP values (figure 1). A generalized additive mixed model with a scaled *t*-distribution was used to analyse the relationship between the amount of light pollution and the date of bud-burst. Budburst date, quantified as the number of days from 1st January in the corresponding year, was incorporated into the model as the response variable and was assumed to follow a *t*-distribution (a symmetric distribution like the Gaussian but with heavier tails). The mean of the response *μ* was modelled in terms of additive influences from the various predictors; specifically, smooth non-parametric functions of the DMSP value, mean spring air temperature and their interaction. Calendar year was incorporated into the model as a random effect to account for inter-annual variation of budburst date. To allow for latitudinal variation in day length and other spatial trends in the data, parametric linear and quadratic terms of Easting, Northing and their interaction were also incorporated additively in the mean of the response. An interaction between DMSP value and temperature was included to analyse whether the relationship between budburst date and DMSP value varied at different temperatures.

The analysis was repeated excluding data points found within large urban areas to remove any residual effect of the UHI not captured by the temperature dataset, along with other potential effects of urbanization on budburst date. The Ordnance Survey Meridian 2 dataset was used to define the boundaries of urban areas, and the analysis was carried out on the data points that fell outside of settlements with a population of greater than or equal to 125 000. As a further check to reduce the possibility of inflated degrees of freedom owing to non-independence of observations in close proximity, the analyses were repeated using only observations that were at least 5 km distant from another observation in the same year. Data from Northern Ireland were also excluded from these further analyses as the Ordnance Survey Meridian 2 dataset does not cover this area.

Predictions from these models, within the limits of the data used for model calibration, were carried out to aid inference. The ‘gam’ function from the R package mgcv (v. 1.8–4) was used for fitting the generalized additive mixed (GAM) models [35,36], and approximate tests of significance were carried out on the model terms by using the function ‘anova.gam’; this function carries out Wald tests of significance on the smooth and parametric terms within a single fitted GAM object. All statistical analyses were carried out using R (v. 64 3.0.3) [37]. See the electronic supplementary material for additional information on statistical methods and model check plots (table 1).

**Table 1.** Terms and properties of generalized additive mixed models fitted to all data.

response variable	explanatory terms				model statistics
*Acer psuedoplatanus* budburst date	smooth terms	EDF	*χ*^2^	*p*-value (approx.)	*R*^2^ (adj*.*)
	DMSP value	0.489	0.299	0.584	0.117
	spring temperature	2.84	183	<0.001	deviance explained
	DMSP value, spring temperature (interaction)	2.10	1.83	0.514	11.8%
	year (random factor)	9.61	389	<0.001	REML
	parametric terms	DF	*χ*^2^	*p*-value	48 517
	Northing	1	10.632	0.00111	no. of obs.
	Easting	1	0.767	0.381	11 968
	Northing : Easting (interaction)	1	6.657	0.00988	
	Northing^2^	1	15.353	<0.001	
	Easting^2^	1	0.358	0.550	
*Fagus sylvatica* budburst date	smooth terms	EDF	*χ*^2^	*p-*value (approx.)	*R*^2^ (adj*.*)
	DMSP value	0.00145	1190.5	<0.001	0.185
	spring temperature	2.885	413.6	<0.001	deviance explained
	DMSP value, spring temperature (interaction)	0.000101	349.6	<0.001	18.2%
	year (random factor)	9.190	542.5	<0.001	REML
	parametric terms	DF	*χ*^2^	*p*-value	39 638
	Northing	1	0.143	0.705	no. of obs.
	Easting	1	76.0	<0.001	10 061
	Northing : Easting (interaction)	1	14.8	<0.001	
	Northing^2^	1	4.15	0.0415	
	Easting^2^	1	9.62	<0.001	
*Quercus robur* budburst date	smooth terms	EDF	*χ*^2^	*p*-value (approx.)	*R*^2^ (adj*.*)
	DMSP value	0.000284	7093.8	<0.001	0.345
	spring temperature	2.955	1111.8	<0.001	deviance explained
	DMSP value, spring temperature (interaction)	0.000155	967.6	<0.001	33.1%
	year (random factor)	9.619	598.9	<0.001	REML
	parametric terms	DF	*χ*^2^	*p*-value	32 971
	Northing	1	8.64	0.00329	no. of obs.
	Easting	1	98.02	<0.001	8908
	Northing : Easting (interaction)	1	58.86	<0.001	
	Northing^2^	1	7.09	0.00775	
	Easting^2^	1	14.10	<0.001	
*Fraxinus excelsior* budburst date	smooth terms	EDF	*χ*^2^	*p*-value (approx.)	*R*^2^ (adj*.*)
	DMSP value	0.00338	953.2	<0.001	0.209
	spring temperature	2.82	313.6	<0.001	deviance explained
	DMSP value, spring temperature (interaction)	0.000133	956.8	<0.001	20.5%
	year (random factor)	9.182	1265.7	<0.001	REML
	parametric terms	DF	*χ*^2^	*p*-value	43 587
	Northing	1	8.99	0.00272	no. of obs.
	Easting	1	10.7	0.001	10 899
	Northing : Easting (interaction)	1	38.6	<0.001	
	Northing^2^	1	9.57	0.002	
	Easting^2^	1	1.51	0.219	

As a test that any observed relationship between DMSP and budburst date was robust to different forms of analysis, for each species we carried out a partial regression on the residuals from a GAM of DMSP (response) and spring temperature (predictor) and the residuals from a GAM of budburst date (response) and spring temperature (predictor). Full results are shown in the electronic supplementary material.

## 3. Results

**Figure 2.**
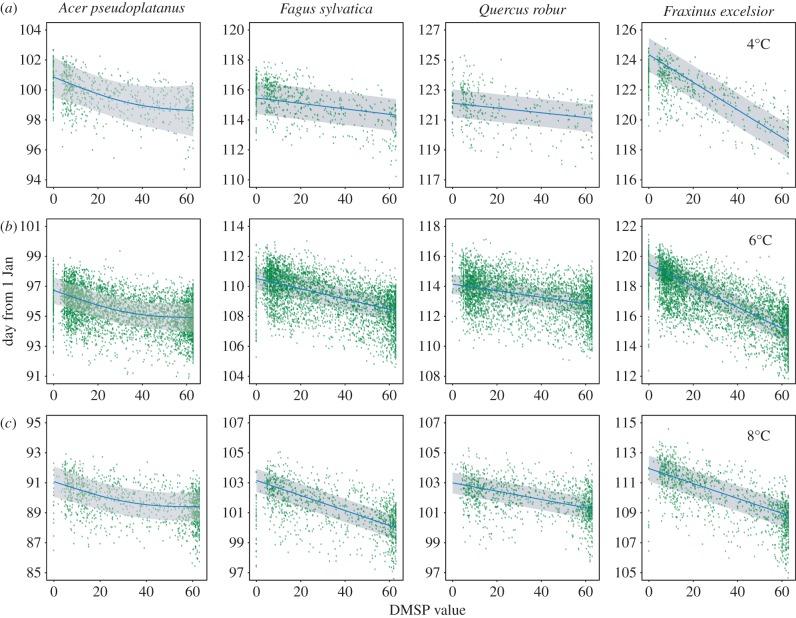
Plotted model predictions, within the bounds of the experimental data used for model calibration, of the relationship between DMSP night-time lights and budburst date at different spring temperatures; 4°C (*a*), 6°C (*b*) and 8°C (*c*), and from left to right (in order of budburst), *Acer*, *Fagus*, *Quercus* and *Fraxinus*. Predictions are made for budburst at the mean latitude of data points included in the model. The blue line represents the predicted mean and the shaded grey area the predicted 95% CIs. Points represent residuals of individual data points where the spring temperature lies within 0.5°C of the prediction temperature in each panel.

**Figure 3.**
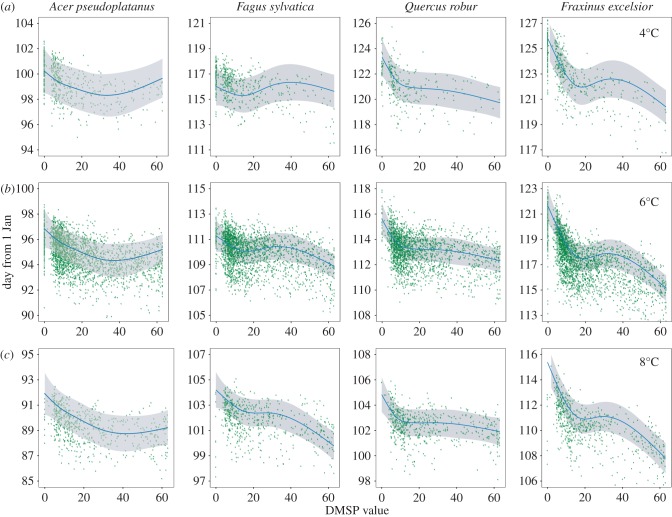
(*a*–*c*) As for figure 2, but with urban areas (populations exceeding 125 000) removed.

Our analysis showed no significant effect of DMSP value on the species with earliest budburst, *A. pseudoplatanus* but significant effects of the DMSP value on budburst date in three of the four species; listed here in order of budburst, *Fa. sylvatica* (*χ*^2^ = 1190.5, *p* ≤ 0.001, *n* = 10 061, *Q. robur* (*χ*^2^ = 7093.8, *p* ≤ 0.001, *n* = 8908) and *Fr. excelsior* (*χ*^2^ = 953.2, *p* ≤ 0.001, *n* = 10 899). In all three cases, the relationship was negative (areas with brighter lights typically experienced earlier bud-burst) and there was a significant interaction between temperature and DMSP (figure 2). The largest magnitude of effect was for *Fr. excelsior* at lower temperatures, where the difference in fitted model predictions between the darkest rural and most brightly lit urban sites was 7 days (figure 2). When large urban areas are excluded from the analysis, the predictions show a qualitatively similar, but nonlinear relationship between budburst date and the DMSP value, and the effect of DMSP value was here also significant for *A. pseudoplatanus* (figure 3 and table 2; *χ*^2^ = 17.73, *p* ≤ 0.001, *n* = 6053). For *Fa. sylvatica*, the effect of DMSP was non-significant, but there was a significant interaction with spring temperature (*χ*^2^ = 433.5, *p* ≤ 0.001, *n* = 6053), with earlier budburst only associated with artificial light at higher temperatures. Both *Q. robur* and *Fr. excelsior* had significant relationships between budburst date and DMSP (*Q. robur χ*^2^ = 8.63, *p* *=* 0.032, *n* = 5296 and *Fr. excelsior χ*^2^ = 37.4, *p* ≤ 0.001, *n* = 6762), and also significant interaction terms with temperature (table 2 and figure 3). Excluding observations from urban areas from the analysis, *Fr. excelsior* buds in areas with average spring temperatures of 4°C are likely to burst approximately 5 days earlier in the brightest areas compared with the darkest areas, and buds that experience average spring temperatures of 8°C are predicted to burst approximately 7.5 days earlier in the brightest areas. As a conservative test for spatial non-independence, further excluding all observations in the close vicinity (within 5 km) of any other observations recorded in the same year had no qualitative effect on the significance levels of the results reported here (see the electronic supplementary material).

**Table 2.** Terms and properties of generalized additive mixed models fitted to data excluding data points found within large urban areas (population ≥12 500).

response variable	explanatory terms				model statistics
*Acer psuedoplatanus* budburst date	smooth terms	EDF	*χ*^2^	*p-*value (approx.)	*R*^2^ (adj*.*)
	DMSP value	2.48	17.73	<0.001	0.121
	spring temperature	2.81	100.43	<0.001	deviance explained
	DMSP value, spring temperature (interaction)	0.00160	224.73	<0.001	12.1%
	year (random factor)	9.98	257.39	<0.001	REML
	parametric terms	DF	*χ*^2^	*p*-value	28 572
	Northing	1	13.525	<0.001	no. of obs.
	Easting	1	3.623	0.0570	7024
	Northing : Easting (interaction)	1	3.750	0.0528	
	Northing^2^	1	26.231	<0.001	
	Easting^2^	1	1.679	0.195	
*Fagus sylvatica* budburst date	smooth terms	EDF	*χ*^2^	*p-*value (approx.)	*R*^2^ (adj*.*)
	DMSP value	2.574	3.03	0.36	0.191
	spring temperature	2.864	276.9	<0.001	deviance explained
	DMSP value, spring temperature (interaction)	0.003	433.5	<0.001	19.1%
	year (random factor)	9.295	325.5	<0.001	REML
	parametric terms	DF	*χ*^2^	*p-*value	23 708
	Northing	1	0.216	0.642	no. of obs.
	Easting	1	95.857	<0.001	6053
	Northing : Easting (interaction)	1	19.916	<0.001	
	Northing^2^	1	2.868	0.0904	
	Easting^2^	1	18.337	<0.001	
*Quercus robur* budburst date	smooth terms	EDF	*χ*^2^	*p*-value (approx.)	*R*^2^ (adj*.*)
	DMSP value	2.62	8.63	0.032	0.360
	spring temperature	2.91	580.3	<0.001	deviance explained
	DMSP value, spring temperature (interaction)	0.000543	86.27	<0.001	33.8%
	year (random factor)	9.891	394.2	<0.001	REML
	parametric terms	DF	*χ*^2^	*p-*value	19 686
	Northing	1	7.68	0.00557	no. of obs.
	Easting	1	12.8	<0.001	5296
	Northing : Easting (interaction)	1	39.4	<0.001	
	Northing^2^	1	6.18	0.0129	
	Easting^2^	1	0.020	0.887	
*Fraxinus excelsior* budburst date	smooth terms	EDF	*χ*^2^	*p-*value (approx.)	*R*^2^ (adj*.*)
	DMSP value	2.61	37.4	<0.001	0.229
	spring temperature	2.52	191.0	<0.001	deviance explained
	DMSP value, spring temperature (interaction)	0.000830	1371.7	<0.001	22.4%
	year (random factor)	9.08	874.9	<0.001	REML
	parametric terms	DF	*χ*^2^	*p-*value	26 950
	Northing	1	4.70	0.0302	no. of obs.
	Easting	1	0.775	0.379	6762
	Northing : Easting (interaction)	1	23.6	<0.001	
	Northing^2^	1	6.71	0.00961	
	Easting^2^	1	12.0	<0.001	

## 4. Discussion

The results highlight, for the first time, to our knowledge, and at a national scale, a relationship between the amount of artificial night-time light and the date of budburst in deciduous trees. This relationship is unlikely to be caused by the UHI effect, as it is robust to the exclusion of large urban areas where temperatures are known to be elevated. Similarly, this effect is unlikely to be related to an increase in temperature alone; the maximum magnitude of effect size predicted between the brightest and darkest sites (7.5 days) is roughly equivalent to that predicted due to 2°C. Specifically, it has already been shown that urban areas are both brighter (DMSP data have been used as a proxy measure of urban extent [29,30]) *and* warmer (UHI effect [32]) but this is, to our knowledge, the first study explicitly investigating the relationship between the amount of night-time light and budburst while controlling for the temperature increases within urban areas. In summary, similar predictions were obtained from a model fitted to budburst data points found outside of large urban areas suggesting that it is night-time lighting causing the advance in budburst as opposed to other factors which can vary owing to urbanization, such as temperature, humidity, water availability and chemical pollution levels [38–41]. In addition, for trees experiencing average spring temperatures of 4°C, the model predicts that budburst will be advanced by up to 7.5 days in the brightest areas compared to the darkest areas.

The exposure of plants to artificial light at night is highly heterogeneous at a fine scale. Skyglow, diffuse light scattered in the atmosphere from city lights, can illuminate areas of many square kilometres to levels exceeding moonlight, but effects of artificial light on phenology have to date only been recorded as a consequence of direct illumination in the vicinity of light sources, which can be several orders of magnitude brighter [13]. As the spatial data for this study was aggregated to 5 km resolution, and the DMSP data have no direct calibration, the DMSP value for each pixel cannot be easily related to an illuminance or irradiance that any individual tree is exposed to at night. Moreover, even in dark pixels, an individual tree adjacent to a street light may be exposed to bright light, while a tree in a large unlit urban park might be relatively dark despite being located in a bright pixel. However, the DMSP pixel brightness is probably a good indication of the density of outdoor light sources, and hence the probability of any tree within that pixel experiencing a relatively high level of direct illumination; observers recording the first budbreak in three trees in close proximity will therefore be considerably more likely to be recording trees exposed to artificial light in ‘bright’ than ‘dark’ pixels.

Our finding that phenology of woodland tree species may be affected by light pollution, suggests that smaller plants growing below the height of street lights are even more likely to be affected. Such results highlight the need to carry out experimental investigation into the impact of artificial night-time lighting on phenology and species interactions. It also suggests that looking at other aspects of phenology, such as leaf senescence, would be highly worthwhile. Importantly, further studies should also try and take into account differences in light quality such as the specific wavelengths of light generated by different lighting types.

## Supplementary Material

Supporting electronic information for ffrench-Constant et al.

## References

[1] Gaston KJ, Bennie J, Davies TW, Hopkins J 2013 The ecological impacts of night time light pollution: a mechanistic appraisal. Biol. Rev. Camb. Phil. Soc. 88, 912–927. (10.1111/brv.12036) 23565807

[2] Basler D, Körner C 2012 Photoperiod sensitivity of bud burst in 14 temperate forest tree species. Agric. For. Meteorol. 165, 73–81. (10.1016/j.agrformet.2012.06.001)

[3] Smith H 2000 Phytochromes and light signal perception by plants: an emerging synthesis. Nature 407, 585–591. (10.1038/35036500) 11034200

[4] Linkosalo T, Lechowicz MJ 2006 Twilight far-red treatment advances leaf bud burst of silver birch (*Betula pendula*). Tree Physiol. 26, 1249–1256. (10.1093/treephys/26.10.1249) 16815827

[5] Visser ME, Holleman LJ 2001 Warmer springs disrupt the synchrony of oak and winter moth phenology. Proc. R. Soc. Lond. B 268, 289–294. (10.1098/rspb.2000.1363) PMC1088605 11217900

[6] Buse A, Dury SJ, Woodburn RJW, Perrins CM, Good JEG 1999 Effects of elevated temperature on multi-species interactions: the case of Pedunculate oak, winter moth and tits. Funct. Ecol. 13, 74–82. (10.1046/j.1365-2435.1999.00010.x)

[7] Buse A, Good JEG 1996 Synchronization of larval emergence in winter moth (*Operophtera brumata* L.) and budburst in Pedunculate oak (*Quercus robur* L.) under simulated climate change. Ecol. Entomol. 21, 335–343. (10.1046/j.1365-2311.1996.t01-1-00001.x)

[8] Basler D, Körner C 2014 Photoperiod and temperature responses of bud swelling and bud burst in four temperate forest tree species. Tree Physiol. 34, 377–388. (10.1093/treephys/tpu021) 24713858

[9] Bennie J, Kubin E, Wiltshire A, Huntley B, Baxter R 2010 Predicting spatial and temporal patterns of bud-burst and spring frost risk in north-west Europe: the implications of local adaptation to climate. Glob. Change Biol. 16, 1503–1514. (10.1111/j.1365-2486.2009.02095.x)

[10] Phillimore AB, Proios K, O'Mahony N, Bernard R, Lord AM, Atkinson S, Smithers RJ 2013 Inferring local processes from macro-scale phenological pattern: a comparison of two methods. J. Ecol. 101, 774–783. (10.1111/1365-2745.12067)

[11] Feeny P 1970 Seasonal changes in oak leaf tannins and nutrients as a cause of spring feeding by winter moth caterpillars. Ecology 51, 565–581. (10.2307/1934037)

[12] Vitasse Y, Basler D 2013 What role for photoperiod in the bud burst phenology of European beech? Eur. J. For. Res. 132, 1–8. (10.1007/s10342-012-0661-2)

[13] Bennie J, Davie TW, Cruse D, Gaston KJ 2016 Ecological effects of artificial light on wild plants. J. Ecol. 104, 611–620. (10.1111/1365-2745.12551)

[14] Cinzano P, Falchi F, Elvidge CD 2001 The first world atlas of the artificial night sky brightness. Mon. Not. R. Astron. Soc. 328, 689–707. (10.1046/j.1365-8711.2001.04882.x)

[15] Hölker F et al. 2010 The dark side of light: a transdisciplinary research agenda for light pollution policy. Ecol. Soc. 15, 13.

[16] Longcore T, Rich C 2004 Ecological light pollution. Front. Ecol. Environ. 2, 191–198. (10.1890/1540-9295(2004)002%5B0191:ELP%5D2.0.CO;2)

[17] Gaston KJ, Davies TW, Bennie J, Hopkins J 2012 Reducing the ecological consequences of night-time light pollution: options and developments. J. Appl. Ecol. 49, 1256–1266. (10.1111/j.1365-2664.2012.02212.x) 23335816 PMC3546378

[18] Davies TW, Bennie J, Inger R, de Ibarra NH, Gaston KJ 2013 Artificial light pollution: are shifting spectral signatures changing the balance of species interactions? Glob. Change Biol. 19, 1417–1423. (10.1111/gcb.12166) PMC3657119 23505141

[19] Navara KJ, Nelson RJ 2007 The dark side of light at night: physiological, epidemiological, and ecological consequences. J. Pineal Res. 43, 215–224. (10.1111/j.1600-079X.2007.00473.x) 17803517

[20] Davies TW, Bennie J, Gaston KJ 2012 Street lighting changes the composition of invertebrate communities. Biol. Lett. 8, 764–767. (10.1098/rsbl.2012.0216) 22628095 PMC3440964

[21] Neil K, Wu J 2006 Effects of urbanization on plant flowering phenology: a review. Urban Ecosyst. 9, 243–257. (10.1007/s11252-006-9354-2)

[22] Jochner SC, Sparks TH, Estrella N, Menzel A 2012 The influence of altitude and urbanisation on trends and mean dates in phenology (1980–2009). Int. J. Biometeorol. 56, 387–394. (10.1007/s00484-011-0444-3) 21604152

[23] Zhang X 2004 The footprint of urban climates on vegetation phenology. Geophys. Res. Lett. 31, L12209 (10.1029/2004GL020137)

[24] Imhoff ML, Tucker CJ, Lawrence WT, Stutzer DC 2000 The use of multisource satellite and geospatial data to study the effect of urbanization on primary productivity in the United States. IEEE Tran. Geosci. Remote Sensing 38, 2549–2556. (10.1109/36.843042)

[25] Zhang X, Friedl MA, Schaaf CB, Strahler AH 2004 Climate controls on vegetation phenological patterns in northern mid- and high-latitudes inferred from MODIS data. Glob. Change Biol. 10, 1133–1145. (10.1111/j.1365-2486.2004.00784.x)

[26] White MA, Nemani RR, Thornton PE, Running SW 2002 Satellite evidence of phenological differences between urbanized and rural areas of the eastern United States deciduous broadleaf forest. Ecosystems 5, 260–273. (10.1007/s10021-001-0070-8)

[27] Roetzer T, Wittenzeller M 2000 Phenology in central Europe: differences and trends of spring phenophases in urban and rural areas. Int. J. Biometeorol. 44, 60–66. (10.1007/s004840000062) 10993559

[28] Baugh K, Elvidge CD, Ghosh T, Ziskin D 2010 Development of a 2009 stable lights product using DMSP-OLS data. Proc. Asia-Pacific Adv. Netw. 30, 114–130. (10.7125/APAN.30.17)

[29] Imhoff ML, Lawrence WT, Elvidge CD, Stutter DC 1997 A technique for using composite DMSP/OLS ‘city lights‘ satellite data to map urban area. Remote Sensing Environ. 61, 361–370. (10.1016/S0034-4257(97)00046-1)

[30] Sutton PC 2003 A scale-adjusted measure of ‘Urban sprawl’ using nighttime satellite imagery. Remote Sensing Environ. 86, 353–369. (10.1016/S0034-4257(03)00078-6)

[31] Bennie J, Davies TW, Duffy JP, Inger R, Gaston KJ 2014 Contrasting trends in light pollution across Europe based on satellite observed night time lights. Sci. Rep. 4, 3789 (10.1038/srep03789) 24445659 PMC3896907

[32] Arnfield AJ 2003 Two decades of urban climate research: a review of turbulence, exchanges of energy and water, and the urban heat island. Int. J. Climatol. 23, 1–26. (10.1002/joc.859)

[33] Perry M, Hollis D 2005 The generation of monthly gridded datasets for a range of climatic variables over the UK. Int. J. Climatol. 25, 1041–1054. (10.1002/joc.1161)

[34] Sparks TH, Carey PD 1995 The responses of species to climate over two centuries: an analysis of the Marsham phenological record. J. Ecol. 83, 321–329. (10.2307/2261570)

[35] Wood SN 2006 Generalized additive models: an introduction with R. London, UK: Chapman and Hall.

[36] Wood SN 2011 Fast stable restricted maximum likelihood and marginal likelihood estimation of semiparametric generalized linear models. J. R. Stat. Soc. 73, 3–36. (10.1111/j.1467-9868.2010.00749.x)

[37] R Core Team. 2014 *R: a language and environment for statistical computing*. Vienna, Austria: R Core Team.

[38] Jochner S, Alves-Eigenheer M, Menzel A, Morellato LPC 2013 Using phenology to assess urban heat islands in tropical and temperate regions. Int. J. Climatol. 33, 3141–3151. (10.1002/joc.3651)

[39] Honour SL, Bell JNB, Ashenden TW, Cape JN, Power SA 2009 Responses of herbaceous plants to urban air pollution: effects on growth, phenology and leaf surface characteristics. Environ. Pollut. 157, 1279–1286. (10.1016/j.envpol.2008.11.049) 19117655

[40] Peñuelas J, Filella I, Zhang X, Llorens L, Ogaya R, Lloret F, Comas P, Estiarte M, Terradas J 2004 Complex spatiotemporal phenological shifts as a response to rainfall changes. New Phytol. 161, 837–846. (10.1111/j.1469-8137.2003.01003.x) 33873715

[41] Kozlov MV, Eränen JK, Zverev VE 2007 Budburst phenology of white birch in industrially polluted areas. Environ. Pollut. 148, 125–131. (10.1016/j.envpol.2006.10.038) 17175079

