# Supplementary material for: Light pollution is associated with earlier tree budburst across the United Kingdom
Source: Proc Biol Sci. 2016 Jun 29;283(1833):20160813. doi: 10.1098/rspb.2016.0813 (PMC4936040; doi:10.1098/rspb.2016.0813)
Supplement: Supporting electronic information for ffrench-Constant et al. [file rspb20160813supp1.docx]

**Supporting information for ffrench-Constant *et al*.**

**Light pollution advances tree budburst across the United Kingdom**

**Notes on model selection**

A generalised additive mixed model (gam) from the mgcv package (v. 1.8-4) were chosen to analyse the data as it allowed us to fit a model with a scaled t-distribution (family=scat), which greatly improved model fit in comparison to a Gaussian distribution. Year was incorporated into the model as a random effect using the (…, bs="re") term (“smooth.construct.re.smooth.spec”). The interaction between the DMSP light pollution value and the average spring temperature was included by using tensor product (ti) smooth terms. See (Wood, 2014) for details on the methodology described above.


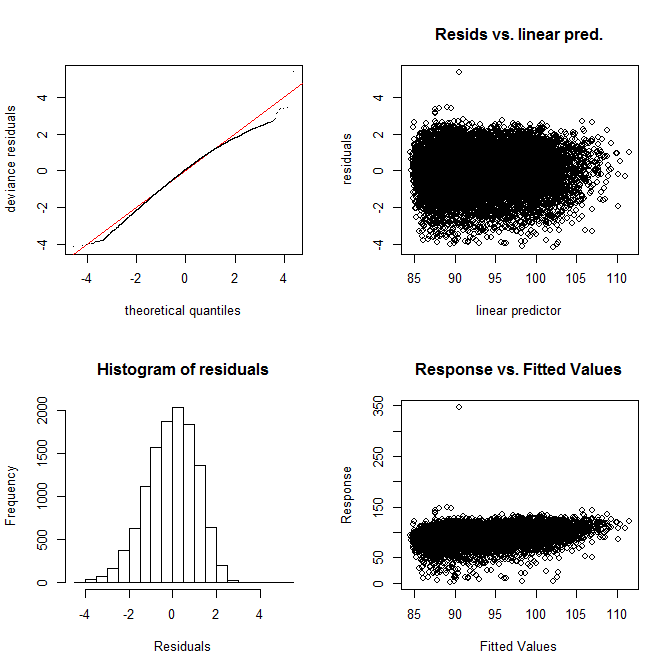

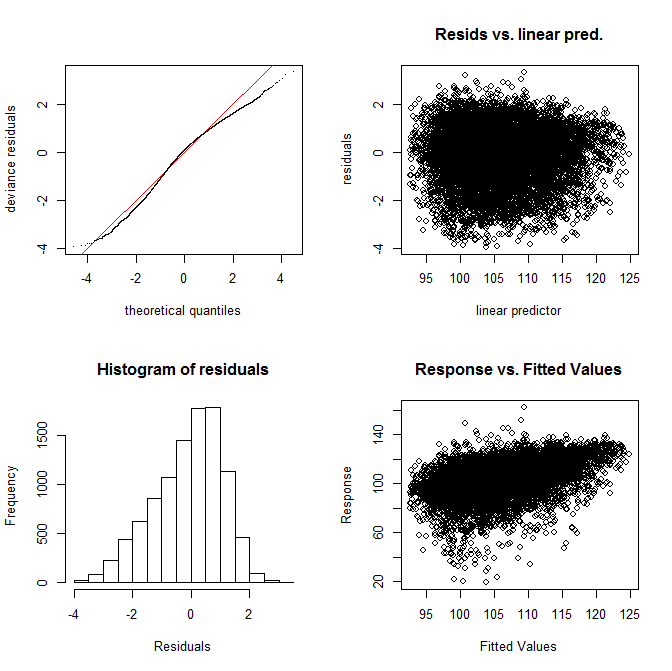

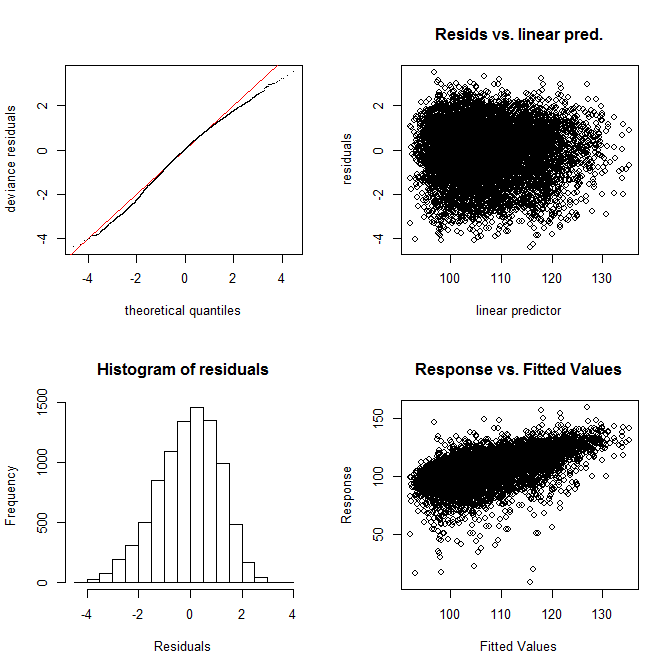

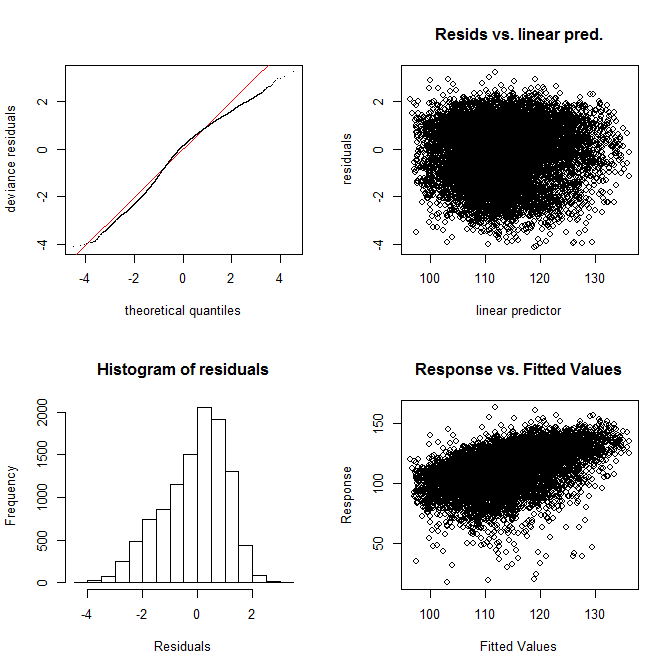


**Figure S1:** Model check plots from model fit to entire datasets, in order from top – sycamore, beech, oak and ash. These plots are the default output from the function “gam.check” in the R package “mgcv”. The QQ plot suggests relatively good model fit, and residual vs fitted plot suggests limited, if any, correlation between the residuals and the fitted values. Also there is evidence of strong linear relationship between the actual data and the fitted values (predictions) implying good model fit.


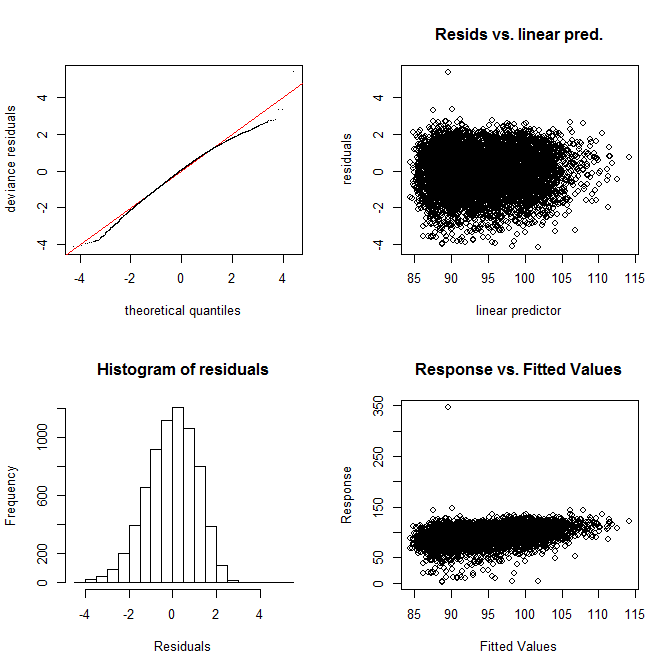

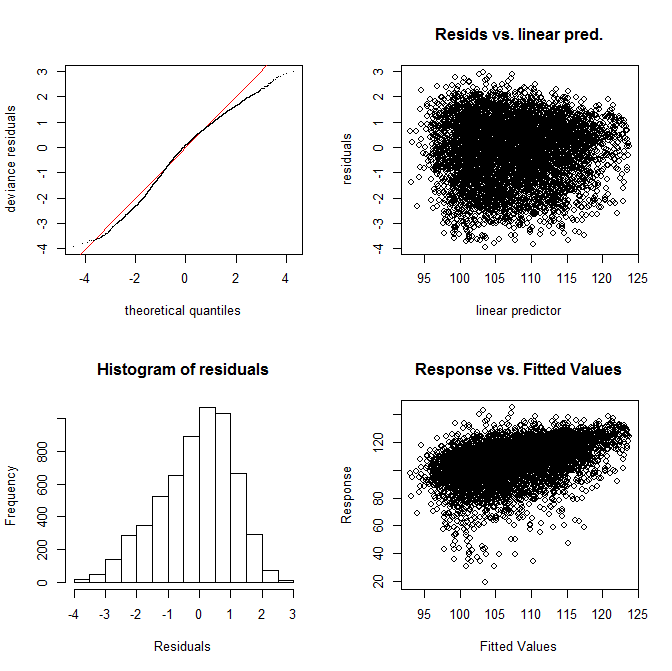

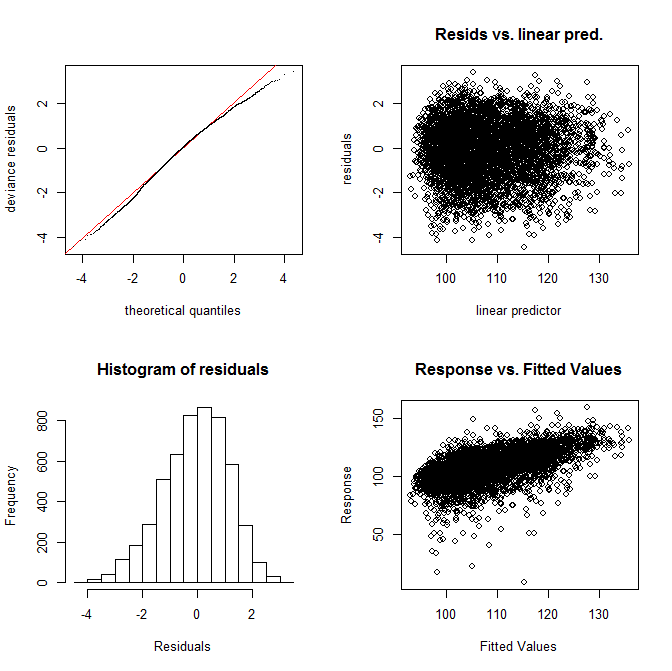


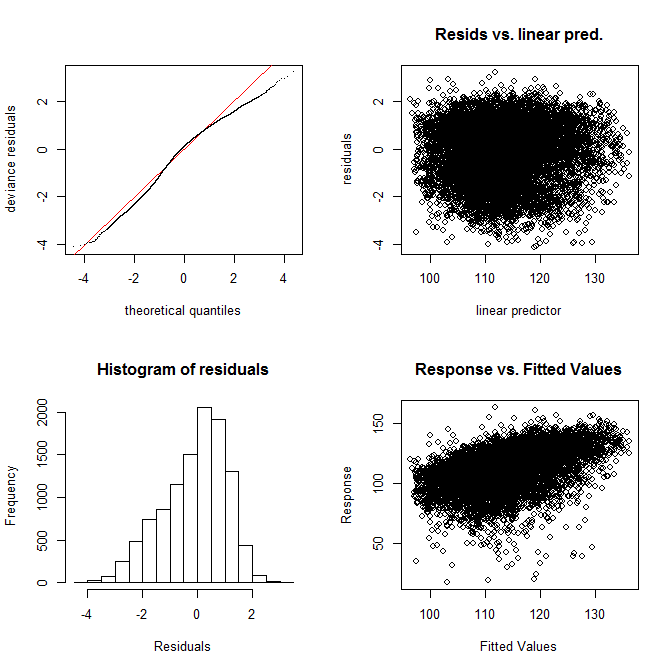


**Figure S2:** Model check plots from model fit to datasets, in order from top – sycamore, beech, oak and ash, as above, but excluding data points from within large urban areas (areas with a population of ≥125,000 excluded). These plots are the default output from the function “gam.check” in the R package “mgcv”. The QQ plot suggests relatively good model fit, and residual vs fitted plot suggests limited, if any, correlation between the residuals and the fitted values. There is also evidence of strong linear relationship between the actual data and the fitted values (predictions) implying good model fit.


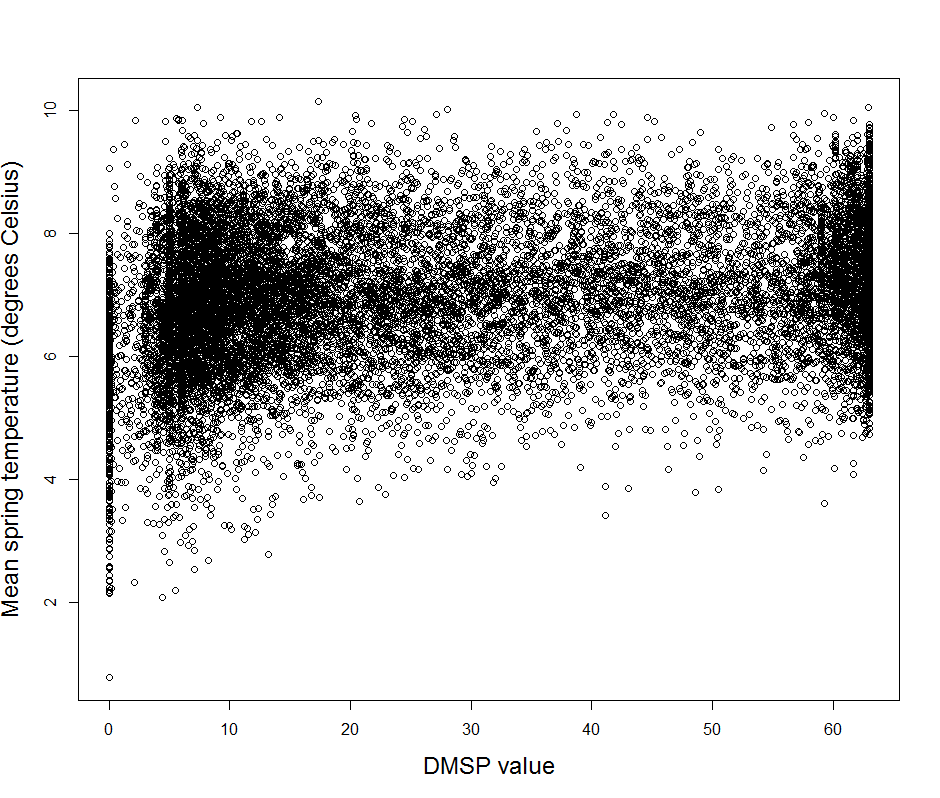


**Figure S4: Scatter plot of intercalibrated DMSP values of observed data points against mean spring temperature (for all species).**

**Notes on partial regression analysis:** To confirm the relationship between DMSP and budburst date independently of temperature, a partial regression analysis was carried out by performing a linear regression on the residuals from a generalised additive model (GAM) of DMSP value against temperature against the residuals of a GAM of budburst day against temperature. The partial regression was highly significant for beech (F-statistic = 66.06, n = 10059, p<0.001), oak (F-statistic = 37.21, n = 8906, p<0.001), and ash (F-statistic = 120.70, n = 10897, p<0.001), but in each case explained a very low proportion of the variance in the dataset (adjusted R^2^ = 0.0065, adjusted R^2^ = 0.0040, adjusted R^2^ = 0.011 respectively). For sycamore, the partial regression was non-significant (F-statistic 2.08, n = 11967, p = 0.150).

**Table S1:** *p*-values for all terms in model runs excluding all records within 5 km of another record in the same year, to test for effects of spatial non-independence. Significance levels are qualitatively similar to those reported in table 2.

| Species | *Acer pseudoplatanus* | *Fagus sylvatica* | *Quercus robur* | *Fraxinus excelsior* |
| --- | --- | --- | --- | --- |
| n | 5775 | 5361 | 4736 | 4600 |
| Deviance explained | 9.45% | 15.80% | 29.30% | 23.30% |
| Northing | <0.001*** | 0.751 | <0.001*** | <0.001*** |
| Easting | <0.001*** | <0.001*** | <0.001*** | <0.001*** |
| Northing2 | <0.001*** | 0.05 | 0.0046** | <0.001*** |
| Easting2 | <0.001*** | <0.001*** | 0.16 | <0.001*** |
| Northing:Easting | <0.001*** | <0.001*** | <0.001*** | <0.001*** |
| DMSP value | <0.001*** | <0.001*** | 0.23 | <0.001*** |
| spring temperature | <0.001*** | <0.001*** | <0.001*** | <0.001*** |
| DMSP:temperature | <0.001*** | <0.001*** | <0.001*** | <0.001*** |
| year | 1 | 0.86 | 0.85 | <0.001*** |

**References**

Wood SN (2014) Package “mgcv” Version 1.8-4.
